# A hospital-based study of survival in oral cancer patients of Tata Memorial Hospital, Mumbai

**DOI:** 10.3332/ecancer.2024.1669

**Published:** 2024-02-15

**Authors:** Monika Lokhande, Sivaranjini Kannusamy, Amey Oak, Sandhya Cheulkar, Shalmali Chavan, Varsha Mishra, Pragati Gode, Aijimol S Thakadiyil, Saket Mendhe, Supriya Kadam, Ganesh Balasubramaniam, Pankaj Chaturvedi, Rajesh Dikshit

**Affiliations:** 1 Homi Bhabha National Institute, Mumbai 400094, India; 2 Division of Cancer Care, Hospital Cancer Registries and Survival Studies, Centre for Cancer Epidemiology, Tata Memorial Centre, Mumbai 400094, India; a https://orcid.org/0009-0005-2587-7188; b https://orcid.org/0009-0004-1893-4191

**Keywords:** oral cancer, sociodemographic factors, clinical factors, registry, survival, India

## Abstract

**Introduction:**

Oral cancer represents a significant global public health concern, with the death rate for lip and oral cavity malignancies experiencing a 1.40-fold increase worldwide in the past three decades. This retrospective study aimed to comprehensively understand overall survival (OS) and the influence of sociodemographic and clinical factors on patients diagnosed with oral cavity cancer.

**Materials and methods:**

The study focused on oral cancer patients enrolled in 2016 and treated at Tata Memorial Hospital, Mumbai, with a follow-up period extending to 5 years until 2021. Utilising the Kaplan–Meier technique and log-rank test, we examined OS and variations based on sociodemographic factors, while the Cox proportional hazard model allowed us to investigate the simultaneous impact of multiple factors on OS.

**Results:**

A total of 1,895 eligible participants were included. The overall 5-year survival rate was 65%. After adjusting for age, gender, education, primary site, tumour grade, TNM staging, treatment intention, status and modality, we found in our study oral cancer patients aged more than 60 years (HR = 1.37, 95% CI: 1.01–1.85, *p*-value 0.03), patients who had poorly differentiated carcinoma (HR = 2.44, 95% CI: 1.56–3.81, *p*-value < 0.001), belonged to stage IV as per TNM staging (HR = 2.44, 95% CI: 1.65–3.61, *p*-value < 0.001), patient who have received partial treatment (HR = 2.44, 95% CI: 1.65–3.61, *p*-value < 0.001) and only chemotherapy (HR = 3.56, 95% CI: 2.43–5.23, *p*-value < 0.001) found to have a higher hazard of dying while literate (HR = 0.73, 95% CI: 0.56–0.95, *p*-value 0.02) are protective.

**Limitations:**

The retrospective nature of the study posed constraints in exploring additional variable associations.

**Implication:**

Overall early detection, appropriate treatment, and regular follow-up are critical for improving the survival rate of patients with oral cavity cancer.

**Conclusion:**

This research proposes that improving the socioeconomic status and promoting proactive treatment-seeking behaviour is crucial for enhancing the survival of oral cancer patients. Cancer hospitals, in collaboration with the wider public healthcare system in India, which includes clinicians and policymakers, should consider these suggestions to enhance cancer treatment and control in low–middle-income countries.

## Introduction

Oral cancer is a serious public health issue globally. The death rate for malignancies of the lip and oral cavity has increased 1.40 times globally over the past three decades. Although smoking is still contributing to up to one-third (30.5%) of worldwide fatalities from this cancer [1]. In 2018, there were 354,864 new instances of oral cancer and 177,384 oral cancer-related deaths worldwide [2]. As per the GLOBOCAN 2020, India has the highest age-standardised incidence rate for oral cancer, with a rate of 9.6 in the South East Asia region [1]. In India, oral cancer ranks in the top three of all cancers and accounts for more than 30% of all cancer cases [2]. Every year, India reports a quarter of all cases worldwide (approximately 77,000 new cases and 52,000 deaths) [3]. South and Southeast Asian countries including India, have the highest rate of oral cancer, as a result, oral cancer prevention is quickly becoming a major global health concern. Survival rates for patients with oral cancer are significantly influenced by sociodemographic and clinical factors [4, 5]. A comprehensive understanding of sociodemographic and clinical characteristics is required to provide targeted treatments, customised treatment programmes and effective public health initiatives. By addressing these traits, healthcare professionals can increase the survival rate of patients with oral cancer by promoting earlier detection, more treatment compliance and easier access to care.

Despite advancements in cancer detection and treatment over the past few decades, 5-year survival rates for oral cancer are still in the 50%–60% range [6, 7]. This study focuses on sociodemographic and clinical parameters to assess their effects on oral cancer patients’ survival rates. This study was conducted at Tata Memorial Hospital (TMH), a top cancer centre in India that offers cancer diagnosis, treatment, education and research. It gives you access to numerous cancer-related investigations, therapies and patient follow-ups. TMH has a homegrown electronic medical record system (EMR), the contents of which include various modules concerning patients’ information entered by clinicians, the modules are shared with patients and providers via the hospital’s intranet and globally via our website. TMH has been carrying out paperless and filmless operations since 2013, enabling the real-time exchange of information and ensuring a continuum of care. This information will be useful for the various research investigations.

TMH has upgraded treatment facilities for patients so that research can determine which treatment is more effective for the patient’s survival. It was useful to comprehend the cancer survival rates by geographic region because at TMH patients often visit from all around India. This study helped us to understand the relationship between sociodemographic and clinical variables on the prognosis of patients with oral cancer. Current data from 2016 was used in this study, and there was a 5-year follow-up from 2016 to 2021.

## Materials and methods

The study was done after the approval from Institute Ethics Committee (IEC). The study included a retrospective review of hospital data from the TMH Cancer Registry, which included all diagnosed oral cancer patients who were recruited at TMH. Oral cancer patients who were recruited at TMH between 1st January 2016 and 31st December 2016, and received treatment at the hospital (TMH). The information was extracted from the patient’s file and the hospital’s EMR. Data collection has been done by using a comprehensive questionnaire that has three sections namely sociodemographic, clinical and follow-up details. Demographic and clinical characteristics such as age, gender, region, occupation, education (a person who cannot read or write is considered as illiterate), marital status, income, initial tumour site, histology of the tumour, grade, location, extent, type of case (new – patients who newly enrolled and received treatment at TMH and old – patients had received treatment outside before coming to TMH) and treatment were noted. Oral cavity subsites were divided into buccal mucosa, tongue, mouth, alveolus, retromolar trigone and hard palate. Surgery, radiation and chemotherapy have all been considered in the treatment analysis. Every 6 months, follow-up was conducted on a regular basis. The follow-up period was commenced from January 2016 till December 2021 for each patient. Follow-up information was collected through the EMR as well as by phone. The follow-up period ended on 31 December 2021. The patients’ overall survival (OS) duration was the period from the date of diagnosis and the date of death or the date of the final follow-up, whichever came first. Overall, 5-year survival was calculated for patients with oral cancer, from January 2016 to December 2021. In addition, the study investigated the influence of sociodemographic and clinical factors on the OS of individuals with oral cancer. The confidentiality of clinical and treatment data was protected. Data entry has been done in-house software. Data analysis was done by using STATA software version 15.0. The mean, standard deviation, median and range, proportion and 95% confidence intervals (CIs) were used to summarise the analytical findings. Kaplan–Meier test and the log-rank test to evaluate OS and variations in survival rates across diverse sociodemographic and clinical factors. Cox proportional hazard models were used to examine the simultaneous impact of various factors on OS in a multifactorial scenario. *p*-value < 0.05 was taken to be significant.

## Results

**Figure 1.**
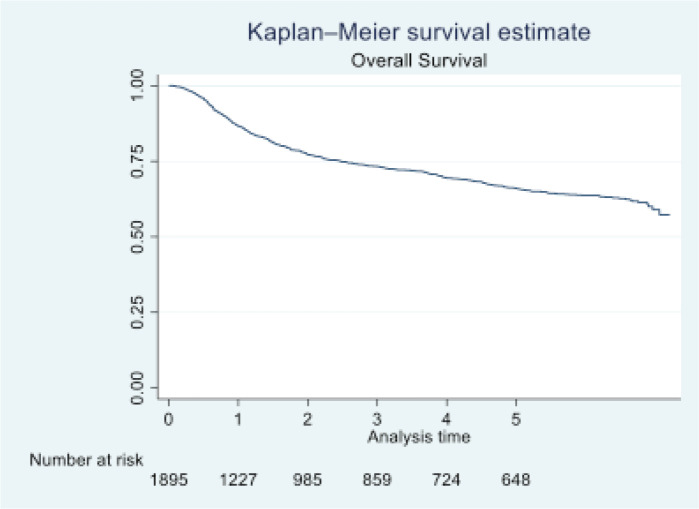
OS of oral cavity cancer patients of TMH, Mumbai, in 2016 (*N* = 1,895).

**Figure 2.**
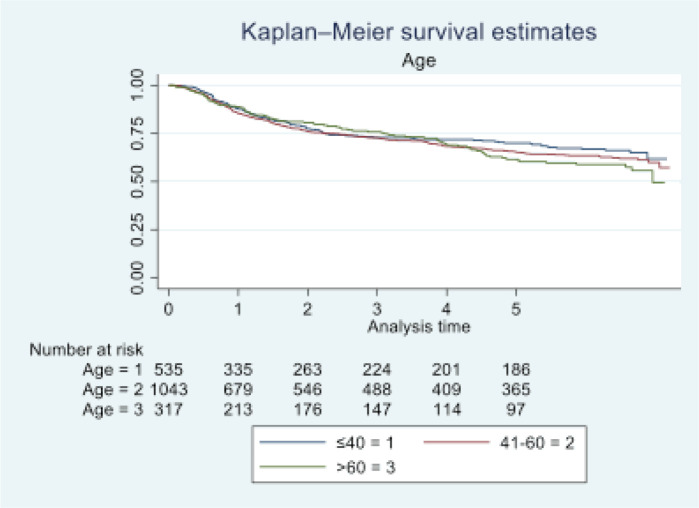
Five-year survival based on the age of oral cancer patients of TMH, Mumbai, in 2016 (*N* = 1,895).

**Figure 3.**
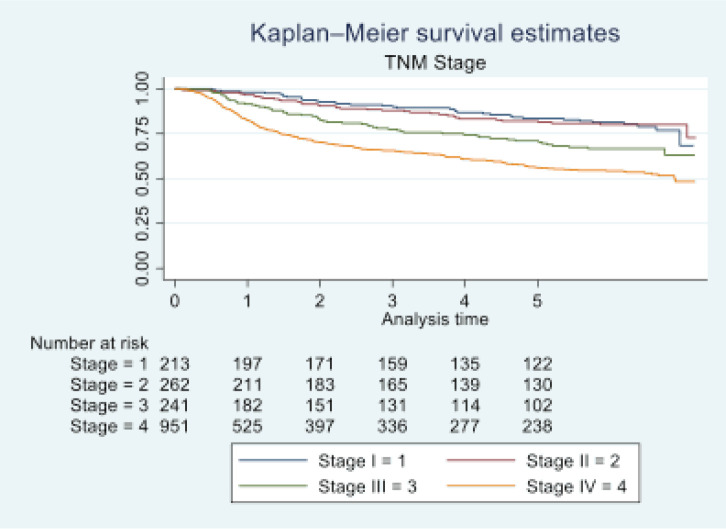
Five-year survival based on TNM stage of oral cancer patients of TMH, Mumbai, in 2016 (*N* = 1,895).

**Figure 4.**
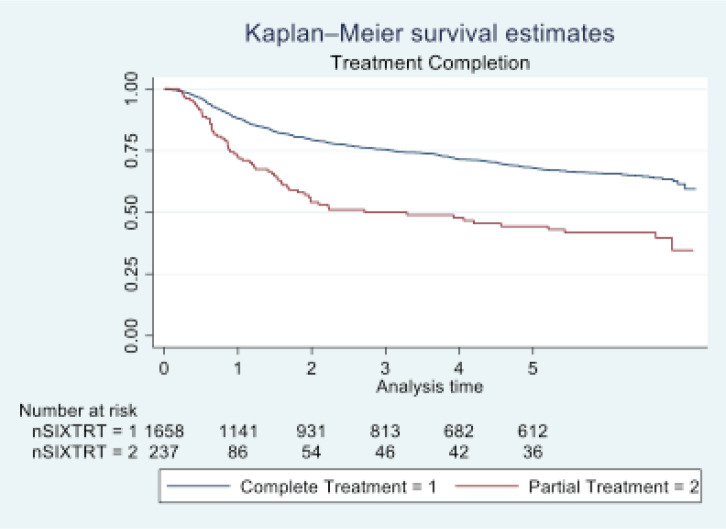
Five-year survival based on treatment completion of oral cancer patients of TMH, Mumbai, in 2016 (*N* = 1,895).

**Figure 5.**
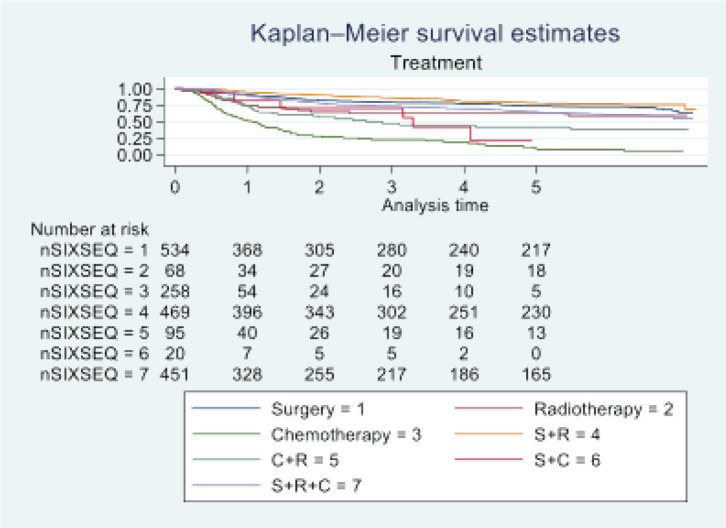
Five-year survival based on the type of treatment of oral cancer patients of TMH, Mumbai, in 2016 (*N* = 1,895).

In 2016, Tata Memorial Centre in Mumbai treated a total of 1,895 cases of oral cancer, with a mean age of 48.36 (11.50). A significant majority of patients (81.74%) were male. Buccal mucosa (C06.0) and tongue (C02) constituted over 70% of cases, while lip (C00) and oral cavity (06.9) were the least common sites. Squamous cell carcinoma was the predominant histology (96.09%). Alarmingly, half of these patients (50.18%) were already at an advanced stage (stage IV). Treatment completion showed that the vast majority (87.49%) completed their treatment, with only 12.51% discontinuing treatment. See Tables 1 and 2 and Figures 1–5.

At the end of the follow-up on 31 December 2021, 24% of the 1,895 patients had passed away, 41% were censored and the overall 5-year survival rate was 65%. Notably, patients aged 40 or younger had a 5-year survival rate (69%), which was unrelated to the stage of illness. Patients in younger age groups (stage I, 64; stage II, 65; stage III, 61 and stage IV, 272) outnumbered those aged 41–60 (65%), and above 60 (60%). The 5-year survival rate for males and females was 65% and 66%, respectively. Site-specific 5-year survival rates varied, with the tongue at 67%, floor of the mouth at 65%, gum and alveolus at 61%, hard palate at 62%, buccal mucosa at 66% and retromolar at 47%. Well-differentiated tumours had a 5-year survival rate of 79%, moderately differentiated at 65%, and poorly and undifferentiated at 58% and 55%, respectively. Stage-wise 5-year survival rates showed a decreasing trend from stage I (83%) to stage IV (55%). Curative patients had a higher 5-year survival rate (68%) compared to palliative patients (11%). Patients who received partial treatment had a significantly lower 5-year survival rate (43%) compared to those who completed treatment which was 67%. The 5-year survival rate for surgery, the most common initial treatment, was 74%, radiotherapy was 58%, and chemotherapy was only 7%. Combination therapies varied, with surgery + radiotherapy having the highest (78%) and surgery + chemotherapy having the lowest (21%) 5-year survival rates, showing significant differences. New patients had a 5-year survival rate of 67%, while old patients had a rate of 50%, and this difference was highly significant (*p*-value < 0.001) (Table 3).

After adjusting for age, gender, education, primary site, tumour grade, TNM staging, treatment–intention, status and modality, we found in our study oral cancer patients aged more than 60 years (HR = 1.37, 95% CI: 1.01–1.85, *p-*value 0.03), patients who had poorly differentiated carcinoma (HR = 2.44, 95% CI: 1.56–3.81, *p*-value < 0.001), belonged to stage IV as per TNM staging (HR = 2.44, 95% CI: 1.65–3.61, *p*-value < 0.001), patient who have received partial treatment (HR = 2.44, 95% CI: 1.65–3.61, *p*-value < 0.001) and only chemotherapy (HR = 3.56, 95% CI: 2.43–5.23, *p*-value < 0.001) found to have a higher hazard of dying while literate (HR = 0.73, 95% CI: 0.56–0.95, *p-*value 0.02), cancer involving buccal mucosa sites (HR = 0.79, 95% CI: 0.62–1.02, *p*-value 0.07) and patient who had the curative intention of treatment 0.56 (HR = 0.56, 95% CI: 0.37–0.85, *p*-value 0.008) had a better prognosis.

## Discussion

This study, conducted at TMH, Mumbai in 2016, aims to determine the 5-year survival rates among individuals diagnosed with oral cancer. The research also investigates sociodemographic and clinical prognostic factors influencing oral cancer survival. Among the 1,895 registered oral cancer patients, 24% succumbed within 5 years and 41% were lost to follow-up. The OS rates in our study for the first, third and fifth years were 86%, 73% and 65%, respectively. In comparison to Yeole *et al*’s [8] findings, our study shows higher survival rates, potentially attributed to differences in data sources. Yeole *et al*’s [8] study included cases from the Mumbai cancer registry, incorporating data from death certificates with broader criteria. In contrast, our analysis focuses exclusively on patients registered and treated at TMH. The OS rate in Zanoni *et al*’s [9] study, which considered various factors, closely aligns with our findings. Listl *et al*’s [10] study reports a lower 5-year relative survival rate of 54.6%, and Kowalski *et al* [11] found a 51.7% OS rate in their research on oral squamous cell carcinoma patients from 2001 to 2012, both lower than our study’s rates.

According to our findings, individuals under 40 exhibited a higher 5-year survival rate (69%) compared to those over 60 (60%). In a study by Ansarin *et al* [12] at the European Institute of Oncology, they investigated 577 consecutive patients. Notably, advanced tongue cancer demonstrated lower fatality rates among younger individuals than their older counterparts, aligning with our study’s outcomes. In a study by Lin *et al* [13], 85 patients over the age of 70 who underwent surgery for early-stage oral cavity squamous cell carcinoma (OCSCC) were retrospectively examined. Disease-free survival and OS were estimated, revealing that elderly individuals with early-stage OCSCC may experience disease progression after surgery. In our study, the hazard ratio indicated that females (0.98) had a slightly more protective effect than males. In terms of education, the majority of patients in our study were literate. Those with some form of education had a 5-year survival rate of 66%, while illiterate individuals had a rate of 61%. Mathew *et al* [14] found varying survival rates based on literacy levels, with illiterate/primary, middle school and secondary school and above groups having rates of 42%, 35% and 48%, respectively. Income status was divided into high, medium and low categories, with corresponding 5-year survival rates of 66%, 62% and 66%. Goswami *et al* [15] noted a correlation between oral cancer patients and income, with a high incidence of catastrophic health expenses reported due to the cost of treatment.

The 5-year survival rate for tongue cancer was 67%, while buccal mucosa cancer had a 5-year survival rate of 66%. Comparative studies indicate varying 5-year survival rates for tongue cancer, ranging from 50% to 68%, with our study aligning closely with the higher end of this spectrum [16, 17]. Bobdey *et al* [18] found a significantly lower 5-year survival rate of 54.01% for buccal mucosa patients in their retrospective analysis of 409 cases. Examining primary site histology, 96% of our study’s patients had squamous cell carcinoma, with a 65% 5-year survival rate. In contrast, Bakshi *et al* [19] reported a lower 5-year survival rate of 58.3% for OCSCC. Fan *et al*’s [20] study on young Chinese patients with OCSCC showed a higher 5-year survival rate of 75%. Poorly differentiated tumours in our study exhibited lower 5-year survival rates (58%) compared to well-differentiated (79%) and moderately differentiated (65%) tumours. Low survival rates for poorly differentiated tumours were also discovered in other research studies [21–24]. Regarding the extent of the disease, our study revealed a 5-year survival rate of 56% for localised cases and 59% for loco-regional cases. However, the study had limited data on distant metastasis cases, making it challenging to determine OS for this group. Yeole *et al* [8] reported lower 5-year survival rates for distant metastases (1.6%) compared to our findings. Analysing survival based on TNM staging, our study demonstrated decreasing survival rates from stage I (83%) to stage IV (55%). This trend aligns with international findings, including a multicentre analysis reporting similar stage-wise survival percentages [25]. The combination of surgery and radiotherapy in our study was associated with the highest 5-year survival rate (78%), while chemotherapy alone had the lowest rate (7%). This aligns with existing research, emphasising the positive impact of surgery and radiotherapy on survival rates [26]. Clinical characteristics in oral cancer, such as primary site, histology, stage and treatment methods, have important implications for public health measures as well as individual patient care. TNM and tumour stage classification were also more significant prognostic variables for these patients’ survival, helping to identify high-risk groupings and guiding therapy. A comprehensive strategy that incorporates prevention, early identification, personalised therapy and supportive care is essential for increasing OS rates and improving the quality of life among individuals impacted by oral cancer.

Strengths of our research include a diverse patient population from across India, enhancing the generalisability of our findings. However, limitations include the exclusion of comorbid conditions that could impact survival and a lack of comprehensive data on patients with distant metastasis.

Recommendations from our study, the study revealed that patients who received their complete course of treatment had a considerably higher rate of survival than those who abandoned their treatment in the middle or discontinued it altogether. Healthcare providers can develop educational materials and counselling programmes that help patients understand the importance of completing their oral cancer treatment to improve their survival rates and quality of life.

After a telephone follow-up, we discovered that the patient’s mental and physical health had deteriorated, prompting the need for support services including pain management and mental health services to assist patients in coping with the mental and physical difficulties brought on by cancer therapy. Overall early detection, appropriate treatment and regular follow-up are critical for improving the survival rate of patients with oral cavity cancer.

## Conclusion

We have deduced from this study that age is the predictive factor that really affects the survival of patients with oral cavity cancer. Our study’s findings showed that education significantly influences survival. Factors contributing to this discrepancy may include improved health literacy, access to healthcare information and enhanced ability to navigate healthcare systems effectively among literate individuals. with buccal mucosa primary site patients showing a significant protective effect. Poorly differentiated, TNM stage IV is considered to be hazardous. Treatment also plays a crucial effect in patient survival. Patients who received complete treatment had significantly greater survival rates compared to those who received partial treatment.

## Conflicts of interest

There is no conflict of interest.

## Funding

There is no funding received for this study.

## Informed consent

The database of the hospital-based cancer registry is under the custody of authorised study personnel. The authors have ensured that the user ID and password for accessing the data are protected. The authors ensure that the rights of the patients are not violated in any manner.

## Author contributions

Ms Monika Lokhande has contributed to the design, statistical analysis, data interpretation and writeups of the manuscript. Dr Sivaranjini has been involved in concept, design, statistical analysis and data interpretation. Dr Amey Oak gave knowledgeable direction and oversight to ensure the study’s methodological rigor and write-ups. Sandhya Cheulkar has contributed to data validation and interpretation. To obtain ethical approval, Dr Shalmali Chavan assisted as well as communicated with the Institutional Review Board (IRB). Dr Varsha, Dr Pragati and Dr Aijimol carried out the data collection and coding and Dr Ganesh Balasubramanium and Dr Pankaj Chaturvedi made substantial contributions to the study’s administration and conceptualisation. Dr Rajesh Dikshit has contributed to the concept, design and management, and assisted in drafting the final report.

## Figures and Tables

**Table 1.** Distribution of oral cancer patients by sociodemographic and clinical characteristics.

Variable	Number (%)	Variable	Number (%)
**Age mean age (48.36 (11.50))**		**Education**	
≤40	535 (28.23)	Literate	1,575 (83.11)
41–60	1,043 (55.04)	Illiterate	318 (16.78)
>60	317 (16.73)	**Occupation**	
**Gender**		Unemployed	327 (16.78)
Male	1,549 (81.74)	Employed	1,466 (77.36)
Female	346 (18.26)	Retired	99 (5.22)
**Region**		**Income**	
North	564 (29.76)	High (>30,374)	142 (7.49)
South	12 (0.63)	Medium (11,362–30,374)	330 (17.41)
Central	132 (6.96)	Low (<11,362)	1,399 (73.83)
East	422 (22.27)	0 (income/unknown)	24 (1.27)
West	714 (37.68)	**Type of case**	
North-east	35 (1.85)	New	1,688 (89.08)
Foreign	16 (0.84)	Old	207 (11.03)
**Primary site (ICD-10)**		**TNM staging**	
Lip (C00.9)	5 (0.26)	Stage I	213 (11.24)
Tongue (C02.9)	697 (36.78)	Stage II	262 (13.83)
Gum and alveolus (C03.9)	246 (12.98)	Stage III	241 (12.72)
Mouth (C04.9)	180 (9.50)	Stage IV	951 (50.18)
Hard palate (C05.0)	39 (2.06)	Outside	228 (12.03)
Buccal mucosa (C06.0)	675 (35.62)	Intent of treatment	
Retromolar area (C06.2)	51 (2.69)	Palliative	139 (7.34)
Oral cavity (C06.9)	2 (0.11)	Curative	1,756 (92.66)
**Primary site histology**		**Type of treatment**	
Squamous cell carcinoma	1,821 (96.09)	Surgery	534 (28.18)
Adenocarcinoma	6 (0.31)	Radiotherapy	68 (3.59)
Others	68 (3.59)	Chemotherapy	258 (13.61)
**Histology grade**		Surgery + radiotherapy	469 (24.75)
Well-differentiated	226 (11.93)	Chemo + radiotherapy	95 (5.01)
Moderately differentiated	860 (45.38)	Surgery + chemotherapy	20 (1.06)
Poorly differentiated	263 (13.88)	Surgery + chemo + radiotherapy	451 (23.80)
Undifferentiated	546 (28.81)	**Treatment completion**	
**Clinical extent**		Complete treatment	1,658 (87.49)
Localised	472 (24.91)	Partial treatment	237 (12.51)
Loco-regional	1,197 (63.17)	**Time to treatment initiation**	
Distant metastasis	6 (0.32)	<30 days	809 (47.92)
Recurrence and treated outside	209 (11.03)	>30 days	879 (52.07)

**Table 2.** OS based on sociodemographic and clinical characteristics.

S. no	Status	Number	1 year (%)	3 years (%)	5 years (%)	*p* value
	OS	1,895	86	73	65	
1.	Age (in years)					0.36
	<40	535	87	72	69	
	41–60	1,043	85	72	65	
	>60	317	88	75	60	
2.	Gender					0.89
	Male	1,549	86	73	65	
	Female	346	87	73	66	
3.	Primary site (ICD-10)					0.55
	Tongue (C02.9)	697	88	73	67	
	Gum and alveolus (C03.9)	246	84	73	61	
	Mouth (04.9)	180	90	73	65	
	Hard palate (05.0)	39	78	66	62	
	Buccal mucosa (C06.0)	675	84	72	66	
	Retromolar area (C06.2)	51	88	69	47	
4.	Histology grade					<0.001
	Well-differentiated	226	93	85	79	
	Moderately differentiated	860	89	74	65	
	Poorly-differentiated	263	85	66	58	
	Undifferentiated	546	78	62	55	
5.	TNM stage					<0.001
	Stage I	213	98	89	83	
	Stage II	262	90	87	80	
	Stage III	241	91	76	70	
	Stage IV	951	82	65	55	
6.	Intent of treatment					<0.001
	Curative	1,756	88	75	68	
	Palliative	139	52	16	11	
7.	Treatment completion					<0.001
	Complete treatment	1,658	88	75	67	
	Partial treatment	237	71	48	43	
8.	Treatment type					<0.001
	Surgery	534	95	79	74	
	Radiotherapy	68	72	62	58	
	Chemotherapy	258	51	22	7	
	Surgery + radiotherapy	469	94	86	78	
	Chemo + radiotherapy	95	72	47	38	
	Surgery + chemotherapy	20	70	56	21	
	Surgery + chemo + radiotherapy	451	89	73	64	

**Table 3.** Multivariate analysis of risk factors for OS of patients with oral cavity cancer using Cox regression (*N* = 1,895).

S. no	Variable	Unadjusted (95% CI)	*p*-value	Adjusted hazard ratio (95% CI)	*p*-value
1.	Age in category				
	<40	1.00			
	41–60 >60	1.13 (0.91–1.39)	0.24	1.27 (1.00–1.60)	0.04
		1.20 (0.91–1.57)	0.18	1.37 (1.01–1.85)	0.03
2.	Gender				
	Male	1.00			
	Female	0.98 (0.78–1.23)	0.89	0.90 (0.70–1.17)	0.47
3.	Education				
	Illiterate	1.00			
	Literate	0.84 (0.67–1.06)	0.15	0.73 (0.56–0.95)	0.02
4.	Primary site (ICD-10)				
	Tongue (C02.9)	1.00			
	Mouth (C04.9)	1.10 (0.79–1.52)	0.55	0.83 (0.59–1.18)	0.32
	Gum and alveolus (C03.9)	1.23 (0.94–1.61)	0.12	0.96 (0.71–1.30)	0.82
	Hard palate (C05.0)	1.28 (0.69–2.36)	0.41	0.97 (0.48–1.94)	0.93
	Buccal mucosa (06.0)	1.10 (0.89–1.36)	0.34	0.79 (0.62–1.02)	0.07
	Retromolar (06.2)	1.47 (0.89–2.42)	0.12	1.06 (0.61–1.84)	0.82
5.	Histologic grade				
	Well-differentiated	1.00			
	Moderately differentiated	1.42 (1.00–2.00)	0.04	1.79 (1.20–2.67)	0.004
	Poorly differentiated	2.11 (1.44–3.11)	<0.001	2.44 (1.56–3.81)	<0.001
	Undifferentiated	2.53 (1.78–3.58)	<0.001	2.01 (1.32–3.04)	0.001
6.	TNM staging				
	Stage I	1.00			
	Stage II	1.01 (0.64–1.58)	0.95	1.03 (0.65–1.63)	0.89
	Stage III	1.85 (1.22–2.80)	0.03	1.95 (1.27–3.01)	0.002
	Stage IV	3.09 (2.18–4.37)	<0.001	2.44 (1.65–3.61)	<0.001
7.	Intention of treatment				
	Palliative	1.00			
	Curative	0.17 (0.13–0.23)	<0.001	0.56 (0.37–0.85)	0.008
8.	Type of treatment				
	Surgery	1.00			
	Radiotherapy	1.91 (1.17–3.10)	0.009	1.63 (0.87–3.06)	0.12
	Chemotherapy	7.72 (5.87–10.1)	<0.001	3.56 (2.43–5.23)	<0.001
	Surgery + radiotherapy	0.73 (0.55–0.97)	0.033	0.63 (0.46–0.87)	0.005
	Chemo + radiotherapy	2.96 (2.01–4.36)	<0.001	2.03 (1.27–3.26)	0.003
	Surgery + chemotherapy	3.23 (1.41–7.36)	0.005	2.22 (0.88–5.57)	0.08
	Surgery + chemo + radiotherapy	1.40 (1.08–1.80)	0.009	1.03 (0.76–1.40)	0.80
